# 成人原发免疫性血小板减少症诊断与治疗中国指南（2025年版）

**DOI:** 10.3760/cma.j.cn121090-20251031-00494

**Published:** 2025-12

**Authors:** 中华医学会血液学分会血栓与止血学组

## Abstract

原发免疫性血小板减少症（ITP）是由于免疫耐受破坏导致血小板破坏增加及生成受损的获得性自身免疫性疾病。近年来，在我国人群结构变化和疾病管理水平提高的背景下，成人ITP呈发病年龄升高、发病率增加的趋势，部分患者兼具出血与血栓风险。本指南在2020年版的基础上结合国内外循证医学证据，更新了部分实验室和辅助检查在诊断评估中的应用，完善了紧急处理与初始治疗策略，系统纳入了新型血小板生成素受体激动剂和小分子激酶抑制剂等治疗选择，拓展了后续治疗的联合方案，并更加重视长期管理中血栓风险与生活质量的权衡，为我国成人ITP的规范化和个体化诊疗提供参考。

## Abstract (en)

Primary immune thrombocytopenia（ITP）is an acquired autoimmune disease characterized by impaired immune tolerance, leading to increased platelet destruction and decreased platelet production. In recent years, with population structure changes and disease management improvements in China, adult ITP exhibits a trend toward older age at presentation and increased incidence. Further, a proportion of patients are at risk of both bleeding and thrombosis. Based on its 2020 edition, this guideline integrates evidence-based medical evidence from domestic and international studies, updates the application of selected laboratory and auxiliary tests in diagnostic evaluation, refines strategies for emergency management and initial therapy, and systematically incorporates new treatment options, including thrombopoietin receptor agonists and small molecule kinase inhibitors. Moreover, it broadens combination regimens for subsequent treatment and places greater emphasis on balancing thrombotic risk and health-related quality of life in long-term management, thereby providing a reference for standardized and individualized care of adult ITP in China.

原发免疫性血小板减少症（primary immune thrombocytopenia, ITP）是一种获得性自身免疫性出血性疾病，以无明确诱因的孤立性外周血血小板计数减少为主要特点。根据国内部分地区流行病学调查数据推测，我国ITP年发病率为（3～7）/10万[1]–[3]。60岁以上老年人是高发群体，育龄期女性的发病率略高于同年龄组男性。该病临床表现差异较大，轻者可为无症状的血小板减少或皮肤黏膜出血，重者可出现严重内脏出血甚至致命的颅内出血。老年患者发生致命性出血的风险明显高于年轻患者。部分患者有乏力和（或）焦虑表现。ITP的主要发病机制是机体对血小板抗原的免疫耐受性丢失，导致自身抗体生成、补体系统活化及细胞免疫异常协同参与血小板破坏，并影响巨核细胞的成熟与产板功能，从而引发血小板减少[4]。

中华医学会血液学分会血栓与止血学组分别于2009年、2011年、2012年、2016年对“成人ITP诊断与治疗中国专家共识”进行了4次更新[5]–[8]，2018年、2020年发布了成人ITP诊断与治疗中国指南[9]–[10]。近年来，ITP研究领域十分活跃，新的治疗药物及临床试验结果不断涌现。结合国内外临床研究进展及实际情况，对《成人原发免疫性血小板减少症诊断与治疗中国指南（2020年版）》[10]进行修订，旨在为成人ITP诊治提供最新的临床指导。

一、诊断要点

ITP的诊断仍基于临床排除法，需除外其他原因所致血小板减少。除详细询问病史及全面体格检查外，诊断要点还包括：

1. 至少连续2次血常规检查示血小板计数<100×10^9^/L，外周血涂片镜检血细胞形态无明显异常。

2. 脾脏一般无肿大。

3. 骨髓检查：ITP患者骨髓细胞学特点为巨核细胞增多或正常，伴成熟障碍[11]。

4. 需排除其他继发性血小板减少症：如自身免疫性疾病、甲状腺疾病、淋巴系统增殖性疾病、骨髓增生异常综合征（MDS）、再生障碍性贫血（AA）、巨幼细胞性贫血、恶性血液病、肿瘤浸润、血栓性微血管病、慢性肝病、脾功能亢进、普通变异型免疫缺陷病（CVID）、感染性疾病、疫苗接种等所致继发性血小板减少；血小板消耗性减少；药物性血小板减少；同种免疫性血小板减少；妊娠期血小板减少；先天性/遗传性血小板减少及假性血小板减少等。

5. 诊断ITP的特殊实验室检查：

（1）抗血小板糖蛋白（anti-GP）特异性自身抗体：优先采用血小板膜结合anti-GP抗体检测。阳性结果对诊断抗体介导的免疫性血小板减少症具有较高的特异性，可用于鉴别免疫性与非免疫性血小板减少，但不能区分原发与继发免疫性血小板减少[12]。

（2）血清血小板生成素（TPO）水平测定：有助于ITP（TPO水平正常）和骨髓衰竭性疾病（TPO水平升高）的鉴别诊断[13]。

对疑诊ITP患者推荐的基本评估和特殊实验室检查详见表1。

**表1:** 成人原发免疫性血小板减少症诊断推荐的实验室检查项目及临床意义

检查项目	临床意义
基本评估	
外周血细胞计数、网织红细胞计数	网织红细胞计数有助于合并贫血患者的鉴别诊断
外周血涂片	根据血细胞形态及数目可鉴别多种原因所致血小板减少症
HBV/HCV/HIV血清学	鉴别病毒感染所致血小板减少症
血清IgG/IgA/IgM水平测定（IVIg治疗前）	鉴别普通变异型免疫缺陷病
骨髓检查（细胞形态学、活检、细胞遗传学、流式细胞术免疫分型）	①鉴别AA、MDS、恶性血液病、肿瘤骨髓浸润等所致血小板减少；②用于常规治疗无效患者及脾切除术前疾病重新评估
抗核抗体谱	鉴别继发性免疫性血小板减少症
抗磷脂抗体（aCL、LA、anti-β2GPⅠ）	鉴别抗磷脂综合征
甲状腺功能及抗甲状腺抗体	鉴别甲状腺功能异常相关血小板减少
凝血系列	除外DIC等凝血障碍性疾病
特殊实验室检查	
抗血小板糖蛋白特异性自身抗体	①鉴别非免疫性血小板减少；②用于常规治疗无效患者及脾切除术前疾病重新评估；③指导IVIg治疗
血清TPO水平测定	①鉴别不典型AA、低增生MDS；②用于常规治疗无效患者及脾切除术前疾病重新评估
MDS相关基因靶向测序	适用于对IVIg、糖皮质激素等治疗反应不良患者，以除外克隆造血相关性血小板减少
全外显子、全基因组测序	适用于幼年起病的反复血小板减少患者、有血小板减少症家族史的患者，以除外先天性/遗传性血小板减少症
网织血小板计数或未成熟血小板分数	有助于鉴别不典型AA、低增生MDS
幽门螺杆菌测定	适用于幽门螺杆菌高发地区或存在明显消化系统症状的患者
直接抗人球蛋白试验	适用于贫血伴网织红细胞增高患者，除外Evans综合征
细小病毒B19、EB病毒、巨细胞病毒核酸定量	用于常规治疗无效患者的疾病重新评估

**注** HBV：乙型肝炎病毒；HCV：丙型肝炎病毒；HIV：人类免疫缺陷病毒；IVIg：静脉注射免疫球蛋白；aCL：抗心磷脂抗体；LA：狼疮抗凝物；anti-β2GPⅠ：抗β2糖蛋白Ⅰ抗体；TPO：血小板生成素；MDS：骨髓增生异常综合征；AA：再生障碍性贫血；DIC：弥散性血管内凝血

6. 出血程度分级：应用出血评分系统量化ITP患者出血情况及风险评估。该系统分为年龄和出血症状两个部分（表2）。ITP患者的出血评分＝年龄评分+出血症状评分（取所有出血症状中最高的分值）。

**表2:** 成人原发免疫性血小板减少症出血评分系统

分值	年龄（岁）		皮下出血（瘀点/瘀斑/血肿）		黏膜出血（鼻腔/齿龈/口腔血疱/结膜）			深部器官出血			
	≥65	≥75	头面部	其他部位	偶发、可自止	多发、难止	伴贫血	内脏（肺、胃肠道、泌尿生殖系统）			中枢神经系统
								无贫血	伴贫血	危及生命	
1	√			√							
2		√	√		√						
3						√		√			
5							√		√		
8										√	√

7. 健康相关生活质量（HRQoL）评估：应重视ITP患者的个体化和全程化管理，建议将HRQoL作为成人ITP管理的重要指标，充分尊重患者意愿，定期应用ITP生活质量评估问卷监测HRQoL，并将患者报告结局纳入评估与管理决策[11],[14]。在制定治疗方案时优先选择不良反应少、对生活质量影响小的治疗药物。同时应加强对HRQoL的动态和系统性监测。

二、疾病的分期、分级

依据病程长短，ITP分为以下三期：①新诊断ITP：确诊3个月以内的患者；②持续性ITP：确诊后3～12个月血小板持续性减少的患者，包括未自发缓解和停止治疗后不能维持完全缓解的患者；③慢性ITP：血小板减少持续时间超过12个月的患者。

重症ITP：血小板计数<10×10^9^/L伴活动性出血，或出血评分≥5分。

难治性ITP：指对糖皮质激素、静脉注射免疫球蛋白（IVIg）、促血小板生成药物及利妥昔单抗（RTX）均无效，或脾切除无效或术后复发，进行诊断再评估仍确诊为ITP的患者。

三、治疗原则及方案

本指南根据美国国立临床诊疗指南数据库证据分级系统对证据进行分级及推荐。证据等级定义如下：Ⅰa级：源于随机对照试验结果的meta分析；Ⅰb级：源于≥1个随机对照试验；Ⅱa级：源于≥1个设计良好的对照研究；Ⅱb级：源于≥1个设计良好的类试验研究；Ⅲ级：源于设计良好的非试验描述性研究，如对比性研究、相关研究；Ⅳ级：源于专家委员会报告或权威专家经验。推荐等级标准定义如下：A级：源于Ⅰa、Ⅰb级证据，要求推荐方案论述总体质量好、一致性强，且内容中包含≥1个随机对照研究；B级：源于Ⅱa、Ⅱb、Ⅲ级证据，要求推荐方案进行了较好的非随机化临床研究；C级：源于Ⅳ级证据，推荐内容证据源于专家委员会报告或权威专家临床经验或意见，缺乏临床研究直接证据。

1. 治疗原则：ITP治疗遵循个体化原则，鼓励患者参与治疗决策，兼顾患者意愿，在治疗不良反应最小化基础上提升血小板计数至安全水平，减少出血事件，尽量采用有限期治疗，实现疾病长期缓解，关注患者HRQoL。鉴于ITP患者动静脉血栓发生率高于普通人群，应重视血栓高危人群的早期识别与干预[15]–[17]，临床可依据《原发免疫性血小板减少症合并血栓/栓塞诊断与防治中国专家共识（2023年版）》[18]推荐量表进行个体化风险评估与管理。

（1）对于血小板计数≥30×10^9^/L、无出血表现且不从事增加出血风险工作、无出血风险因素的ITP患者，可予以观察随访。若患者有活动性出血症状（出血症状评分≥2分），不论血小板减少程度如何，都应开始治疗。

（2）增加出血风险因素：①高龄和长ITP病史；②血小板功能缺陷；③凝血障碍；④高血压；⑤外伤或手术；⑥感染；⑦抗血小板、抗凝或非甾体类药物治疗等。

（3）ITP患者部分临床常规操作或手术以及接受药物治疗时，血小板计数参考值[10]：龈上洁治术及深度清洁：≥（20～30）×10^9^/L；拔牙或补牙：≥（30～50）×10^9^/L；小手术：≥50×10^9^/L；大手术：≥80×10^9^/L；神经外科大手术：≥100×10^9^/L；抗血小板或抗凝单药治疗：≥（30～50）×10^9^/L；抗血小板联合抗凝治疗：≥（50～70）×10^9^/L。

2. 紧急治疗：重症ITP患者或非重症患者需要急症手术或有创操作时，应迅速提升血小板计数至安全水平。可给予IVIg 1 g·kg^−1^·d^−1^×1～2 d、静脉甲泼尼龙500～1 000 mg/d×3 d（C级推荐）。上述措施可单用或联合应用，或进一步联合重组人血小板生成素（rhTPO）300～600 U·kg^−1^·d^−1^、罗普司亭/罗普司亭N01 3～10 µg/kg每周皮下注射治疗，并及时予血小板输注（Ⅲ/Ⅳ级证据）。

其他紧急治疗措施包括长春碱类药物、急症脾切除、抗纤溶药物、控制高血压、口服避孕药控制月经过多、停用抗血小板药物等（C级推荐）。

3. 初始治疗

（1）糖皮质激素：

①大剂量地塞米松（HD-DXM）：40 mg/d×4 d，口服或静脉给药，无效或复发患者可在2周内重复1个周期。治疗过程中注意监测血压、血糖水平，注意预防感染及消化道溃疡出血。

②泼尼松（PDN）：1 mg·kg^−1^·d^−1^，最大剂量80 mg/d，分次或顿服，起效后应尽快减量，6～8周内停用，减停后不能维持疗效患者考虑启动后续治疗。如需维持治疗，PDN的安全剂量不宜超过5 mg/d。2周内PDN治疗无效患者应尽快减停。

糖皮质激素依赖：指需要5 mg/d以上PDN或频繁间断应用糖皮质激素维持血小板计数≥30×10^9^/L和（或）避免出血。

HD-DXM治疗7 d内反应率明显高于PDN，但持续反应率（SRR）、严重出血改善无明显差异（Ⅰb级证据）[19]–[20]。高龄及药物控制不佳的糖尿病、高血压、青光眼、消化性溃疡等患者应慎用。应用HD-DXM的同时建议予抗病毒药物，预防疱疹病毒、乙型肝炎病毒（HBV）等再激活（Ⅳ级证据，C级推荐）。长期应用糖皮质激素可出现高血压、高血糖、急性胃黏膜病变等不良反应，部分患者还可出现骨质疏松、股骨头坏死。

注意糖皮质激素对精神健康的影响，定期评估患者治疗期间HRQoL（抑郁、疲劳、精神状态等）。HBV-DNA复制水平较高的患者慎用糖皮质激素，治疗方案的制订可参照《慢性乙型肝炎防治指南（2022年版）》[21]。

（2）IVIg：主要用于：①紧急治疗；②糖皮质激素不耐受或有禁忌证的患者；③妊娠或分娩前。推荐400 mg·kg^−1^·d^−1^×5 d或1 g·kg^−1^·d^−1^×1～2 d。有条件者可行anti-GP特异性自身抗体检测，有助于IVIg的疗效预判[22]。IgA缺乏和肾功能不全患者应慎用。

（3）以糖皮质激素为基础的初始联合治疗：对于新诊断成人ITP患者，激素治疗有效但减停过程中疗效减弱者，可在糖皮质激素基础上联合1种其他药物，包括促血小板生成药物、RTX、奥司他韦、全反式维甲酸（ATRA）、吗替麦考酚酯（MMF）、咖啡酸，以减少糖皮质激素长期应用的不良反应，提高疗效并延长缓解持续时间。目前有设计良好的前瞻性多中心随机对照临床试验（Ⅰb级证据）支持的初始联合治疗方案见表3。

**表3:** 成人原发免疫性血小板减少症初始联合治疗方案

方案	用法用量	疗效
HD-DXM联合rhTPO[23]	DXM 40 mg/d×4 d×1～2个周期；rhTPO 300 U·kg^−1^·d^−1^×14 d	ORR 89.0％，TTR 4 d，6个月SRR 51.0％
HD-DXM联合RTX[24]	DXM 40 mg/d×4 d×1～3个周期；RTX 375 mg/m^2^每周×4周	6个月SRR 63.0％
HD-DXM联合奥司他韦[25]	DXM 40 mg/d×4 d×1～2个周期；奥司他韦150 mg/d（分2次）×10 d	ORR 82.1％，TTR 4 d，6个月SRR 42.8％
HD-DXM联合ATRA[26]	DXM 40 mg/d×4 d×1～2个周期；ATRA 20 mg/d（分2次）×12周	ORR 91.0％，TTR 6 d，6个月SRR 68.0％
HD-DXM联合咖啡酸[27]	DXM 40 mg/d×4 d×1～2个周期；咖啡酸0.9 g（分3次）×12周	ORR 77.8％，TTR 6 d，6个月SRR 56.5％
糖皮质激素联合MMF[28]	泼尼松龙1 mg·kg^−1^·d^−1^，10周内减停，或DXM 40 mg/d×4 d×1～3个周期；MMF 2 g（分2次）×6个月	ORR 91.5％，24个月SRR 78.0％

**注** HD-DXM：大剂量地塞米松；rhTPO：重组人血小板生成素；RTX：利妥昔单抗；ATRA：全反式维甲酸；MMF：吗替麦考酚酯；ORR：总反应率；TTR：起效时间；SRR：持续反应率

4. 后续治疗

（1）促血小板生成药物：包括rhTPO和血小板生成素受体激动剂（TPO-RA）类药物海曲泊帕、艾曲泊帕、罗普司亭N01/罗普司亭、阿伐曲泊帕。此类药物一般1～2周起效，总反应率（ORR）可达60％以上（Ⅰb级证据，A级推荐），增加初始剂量有助于缩短起效时间（TTR）。多数患者停药后不能维持疗效，通常需维持治疗，建议应用最小有效剂量维持患者血小板计数（50～150）×10^9^/L。对于治疗≥6个月且血小板计数≥50×10^9^/L的稳定患者，可尝试逐步减量停药，但需密切随访和监测。TPO-RA类药物减量时建议依据起始剂量调整后续用药剂量或频次：若采用爬坡剂量起步，达到稳定疗效后可优先减少用药频次；若为中或大剂量起步，则可先减少用药剂量（C级推荐）。rhTPO足剂量应用2周、TPO-RA足剂量治疗4周无效时需考虑换药。疗效丢失可能与服药依从性差、感染或产生药物性中和抗体相关，可通过调整用药、积极控制感染、联合增效剂（双醋瑞因）或免疫抑制剂等手段进行干预。不同TPO-RA可相互转换，应答率为60％～87.5％[29]–[30]。应用促血小板生成药物需警惕血栓及骨髓纤维化等不良反应，定期监测相关指标。具体药物使用和管理可参考《促血小板生成药物临床应用管理中国专家共识（2023年版）》[31]。促血小板生成药物在ITP患者中的用法用量、注意事项见表4。

**表4:** 促血小板生成药物治疗成人原发免疫性血小板减少症给药方案

药物	剂量	给药方式	注意事项
rhTPO	300～600 U·kg^−1^·d^−1^［32]	皮下注射	血栓/栓塞风险、生成药物中和性抗体等
海曲泊帕	2.5～7.5 mg/d	空腹顿服	与乳制品、阳离子矿物质补充剂应用间隔>2 h；肝毒性；血栓风险增加；网硬蛋白形成等
艾曲泊帕	25～75 mg/d	空腹顿服	抗酸药、乳制品、阳离子矿物质补充剂使用前间隔>2 h或服药后间隔>4 h服用艾曲泊帕；肝毒性；血栓风险增加；网硬蛋白形成等
罗普司亭N01/罗普司亭	1～10 µg/kg每周	皮下注射	血栓/栓塞风险、网硬蛋白形成、生成药物中和性抗体等
阿伐曲泊帕	20～40 mg/d	随餐顿服	血栓/栓塞风险、网硬蛋白形成等

**注** rhTPO：重组人血小板生成素

（2）RTX：有效率50％左右，长期反应率20％～25％（Ⅱa类证据，B级推荐）[33]。有2种常用给药方案：①标准剂量方案：375 mg/m^2^静脉滴注，每周1次，共4次，通常在首次用药后4～8周内起效。②小剂量方案：100 mg静脉滴注，每周1次，共4次，或375 mg/m^2^静脉滴注1次，TTR略长。目前研究显示，RTX 375 mg/m^2^单次注射方案与低剂量100 mg/周×4周方案在疗效和安全性方面无显著差异，但单次给药可缩短患者住院时间[32]，建议每3周补充免疫球蛋白5～10 g预防感染（C级推荐）。对于乙型肝炎患者的病毒激活预防，参考2024年版《成人乙型肝炎病毒感染筛查、检测及管理专家建议》[34]。

（3）脾酪氨酸激酶（SYK）抑制剂：福坦替尼100 mg口服，每日2次；4周后如血小板计数未升至≥50×10⁹/L，可增至150 mg，每日2次。ORR 43％～73％，TTR约2周，6个月SRR 20％～50％（Ⅰb级证据，A级推荐）；在既往接受TPO-RA、RTX或脾切除治疗并未获益的患者中，仍有近20％可达持续反应（SR）[35]–[36]。常见不良反应包括腹泻、血压升高、肝功能异常等。一项Ⅲ期临床试验中，国产SYK抑制剂索乐匹尼布300 mg/d的ORR为80％，中位TTR 8 d，6个月SRR 48％（14～24周内≥4次随访血小板计数≥50×10^9^/L）[37]。

（4）布鲁顿酪氨酸激酶（BTK）抑制剂：利扎鲁替尼400 mg口服，每日2次，ORR为64％，中位TTR 15 d，24周持续性血小板反应率（后12周内≥8次血小板计数≥50×10⁹/L）23％（Ⅰb级证据，A级推荐）[38]。患者总体耐受性良好，常见不良反应为腹泻、恶心、头痛等。一项Ⅱ期临床试验显示，国产BTK抑制剂奥布替尼的剂量为50 mg/d，ORR为40％，中位TTR 9 d，6个月SRR 30％；既往对糖皮质激素或IVIg有反应的亚组的ORR为75％，提示既往对一线治疗有应答的患者疗效更佳[39]。目前奥布替尼已完成上市前Ⅲ期临床研究，相关疗效与安全性数据尚未正式公开。

（5）FcRn拮抗剂：艾加莫德5～10 mg/kg，每周1次静脉滴注，连续4周，ORR为46％，中位TTR 7 d，38％的患者在随访期内血小板计数≥50×10^9^/L超过10 d。总体耐受性良好，常见不良反应包括头痛、血尿、瘀点等[40]。

（6）地西他滨：3.5 mg·m^−2^·d^−1^×3 d，静脉滴注，间隔3周后再次给药，共3～6个周期，治疗3个周期无效患者停用。ORR约50％，6个月SRR约40％，不良反应轻微[41]（Ⅲ级证据，B级推荐）。

（7）联合治疗：由于ITP发病机制异质性较高，不同药物在起效时间和疗效维持时间方面存在差异。联合治疗通过多机制协同作用，覆盖更多致病靶点，提高初始反应率和SRR。目前有设计良好的前瞻性多中心随机对照临床试验（Ⅰb级证据）支持的后续联合治疗方案见表5。

**表5:** 成人原发免疫性血小板减少症后续联合治疗方案

方案	给药策略	患者人群	疗效
rhTPO联合RTX[42]	rhTPO 300 U·kg^−1^·d^−1^×2周；RTX 100 mg/周×4周	糖皮质激素治疗无效或复发患者	ORR 79.2％，TTR 7 d，6个月SRR 67.2％，12个月SRR 44.3％
RTX联合ATRA[43]	RTX 100 mg/周×6周；ATRA 20 mg·m^−2^·d^−1^×12周	糖皮质激素治疗无效或复发患者	ORR 80％，TTR 28 d，6个月SRR 61％
艾曲泊帕联合双醋瑞因[44]	艾曲泊帕75 mg/d×14 d；双醋瑞因100 mg/d（分2次）×14 d，个体化维持	艾曲泊帕足量治疗2周无效患者	ORR 44％，TTR 6.5 d，6个月SRR 20％
他克莫司联合达那唑[45]	他克莫司0.03 mg·kg^−1^·d^−1^×12周；达那唑400 mg/d（分2次）×12周	糖皮质激素无效患者	ORR 72％，6个月SRR 65％
ATRA联合达那唑[46]	ATRA 20 mg/d（分2次）；达那唑400 mg/d（分2次）×16周	一线治疗无效或复发患者	ORR 82％，TTR 35 d，中位持续应答时间18.2个月

**注** rhTPO：重组人血小板生成素；RTX：利妥昔单抗；ATRA：全反式维甲酸；ORR：总反应率；TTR：起效时间；SRR：持续反应率

（8）新药Ⅱ/Ⅲ期临床试验。

（9）脾切除术：短期有效率为70％～90％，5年持续有效率为40％～50％[47]。适用于：①糖皮质激素、免疫球蛋白或TPO-RA耐药/依赖；②存在药物禁忌证或无法耐受长期治疗不良反应；③患者积极追求脱离药物治疗（需充分知情手术风险）。脾切除应在ITP确诊12个月后进行，术中留意有无副脾，如发现则应一并切除（C级推荐）。切脾前需对ITP的诊断进行重新评估，建议行anti-GP特异性自身抗体、TPO水平、MDS相关基因靶向测序检查。推荐对术后血小板计数上升过高、过快者进行血栓风险评估，对中高危患者予血栓预防治疗（C级推荐）。有条件的患者脾切除2周前可行疫苗接种（肺炎球菌、脑膜炎球菌、流感嗜血杆菌）。

（10）其他治疗药物或方案：硫唑嘌呤、环孢素A、达那唑、长春碱类等。此类药物缺乏足够的循证医学证据，可根据医师经验及患者状况进行个体化选择。

成人ITP诊治流程见图1。

**图1:**
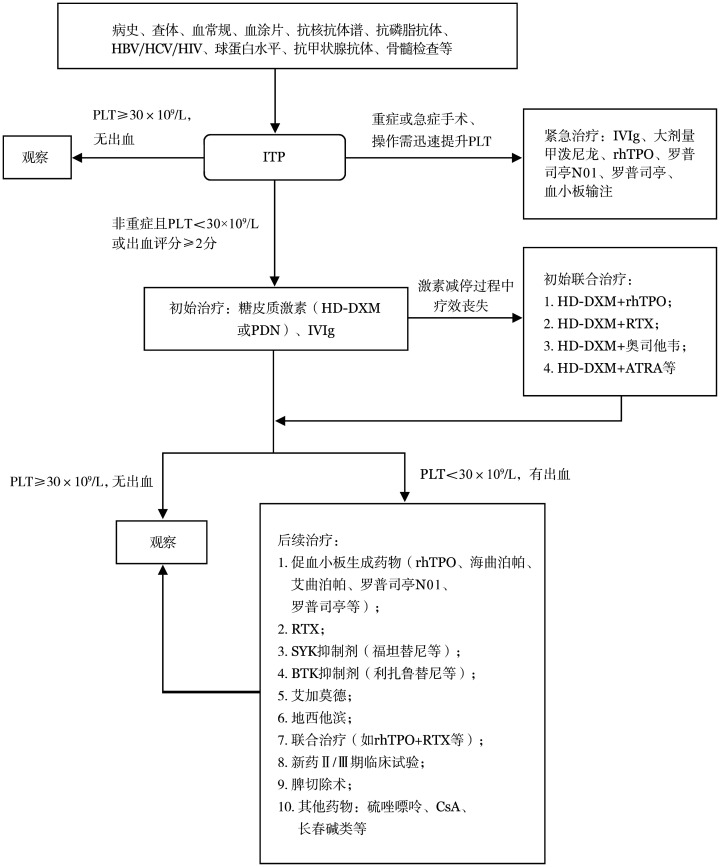
成人原发免疫性血小板减少症（ITP）诊治流程图 **注** HBV：乙型肝炎病毒；HCV：丙型肝炎病毒；HIV：人类免疫缺陷病毒；PLT：血小板计数；ITP：原发免疫性血小板减少症；IVIg：静脉注射免疫球蛋白；rhTPO：重组人血小板生成素；HD-DXM：大剂量地塞米松；PDN：泼尼松；RTX：利妥昔单抗；ATRA：全反式维甲酸；SYK：脾酪氨酸激酶；BTK：布鲁顿酪氨酸激酶；CsA：环孢素A

四、疗效判断

1. 完全反应（CR）：治疗后血小板计数≥100×10^9^/L且无出血表现。

2. 有效（R）：治疗后血小板计数≥30×10^9^/L，较基础血小板计数增加至少2倍，且无出血表现。

3. 无效（NR）：治疗后血小板计数<30×10^9^/L，或血小板计数增加不到基础值的2倍，或有出血。

4. 复发：治疗有效后，血小板计数降至30×10^9^/L以下，或降至不到基础值的2倍，或出现出血症状。

5. 持续有效：患者疗效维持至开始治疗后6个月及以上。

6. 早期反应：治疗开始1周达到有效标准。

7. 初步反应：治疗开始1个月达有效标准。

8. 缓解：治疗开始后12个月时血小板计数≥100×10^9^/L。

在定义CR或R时，应至少检测2次血小板计数，间隔至少7 d。定义复发时至少检测2次血小板计数，间隔至少1 d。
